# Change-point detection using diffusion maps for sleep apnea monitoring with contact-free sensors

**DOI:** 10.1371/journal.pone.0306139

**Published:** 2024-06-27

**Authors:** Momo Shirotori, Yasuo Sugitani

**Affiliations:** 1 Digital Strategy Department, Chugai Pharmaceutical Co., Ltd., Chuo-ku, Tokyo, Japan; 2 Biometrics Department, Chugai Pharmaceutical Co., Ltd, Chuo-ku, Tokyo, Japan; University of South Australia, AUSTRALIA

## Abstract

Monitoring and improving the quality of sleep are crucial from a public health perspective. In this study, we propose a change-point detection method using diffusion maps for a more accurate detection of respiratory arrest points. Conventional change-point detection methods are limited when dealing with complex nonlinear data structures, and the proposed method overcomes these limitations. The proposed method embeds subsequence data in a low-dimensional space while considering the global and local structures of the data and uses the distance between the data as the score of the change point. Experiments using synthetic and real-world contact-free sensor data confirmed the superiority of the proposed method when dealing with noise, and it detected apnea events with greater accuracy than conventional methods. In addition to improving sleep monitoring, the proposed method can be applied in other fields, such as healthcare, manufacturing, and finance. This study will contribute to the development of advanced monitoring systems that adapt to diverse conditions while protecting privacy.

## Introduction

Sleep is an essential component of human health and well-being, and maintaining its quality is an important public health issue [1]. Impaired sleep quality has negative consequences, ranging from chronic health problems to poor performance in daily life [2–7]. Therefore, accurate detection of changes in sleep patterns and early identification of potential disturbances are critical to the development of effective sleep management and intervention strategies. Recently, there has been significant progress in sleep disorder monitoring technologies, and more precise and user-friendly methods have emerged [8, 9]. Conventional polysomnography-based sleep monitoring techniques [10, 11] are invasive and can affect the comfort and privacy of patients; however, a contactless approach using modern sensor technology [12–21] can overcome these challenges. Among the available strategies, detecting change points in time series data plays a central role in the diagnosis of sleep disorders and evaluation of sleep quality. In this study, we propose a new change point detection method that goes beyond conventional methods to capture changes where breathing stops more accurately. It aims to provide insights to improve the quality of sleep and ultimately improve people’s quality of life.

Change-point detection involves identifying the instance at which the behavior of time-series data changes (i.e., change points), such as shifts in mean value or variance. Several methods have been proposed to achieve this, and it has received considerable attention in statistical and machine learning because of its wide range of applications, including quality control [22], climate research [23], and speech recognition [24]. In the classic approach, the change point is captured based on cumulative sum (CUMSUM) statistics [25], Hotelling’s *T*^2^ (H-T2), and *k*-nearest neighbors (KNN) method. CUMSUM statics is widely used today for detecting changes in the mean structure over time as well as in the variance structure [26–28]. Another method, single-spectrum transformation (SST) [29–31], detects the change point using the subspace method with partial time-series data without estimating the density ratio. SST extracts representative waveforms from past and present sub-sequence time series data using the subspace method compares them and detects large changes as change points. As SST performs better for time-series data with periodic changes, it is more effective for time-series sensor data during sleep. However, in the context of change point detection during sleep using contact-free sensors, factors such as environmental noise and sensor drift can influence the readings, potentially leading to false detections or missed change points. Even with the SST method, these types of noise can affect the process of extracting representative waveforms from partial time-series data. Because these representative waveforms are derived via principal component analysis (PCA), a linear dimensionality reduction method, there are limitations to adequately capturing more complex waveforms with nonlinear structures. To address these challenges, it is necessary to develop a change point detection method suitable for sleep monitoring using non-contact sensors. By enhancing the robustness against noise, a more accurate analysis of sleep states can be achieved, thereby improving the quality of sleep monitoring. It is anticipated that these improved methods will contribute to early detection of sleep disorders and enhancement of sleep quality in the future.

In this study, we introduce a sophisticated method that enhances the capabilities of change-point detection beyond the conventional SST approach to address the limitations of existing techniques. We propose a novel change-point detection method that uses diffusion maps [32] (CPD-DM), which is a nonlinear dimensionality-reduction method, based on the SST algorithm [24]. In PCA, the Euclidean distance is used to extract representative waveforms. In diffusion maps, the diffusion distance is utilized to extract waveforms, considering the transition probability from past data. This allows for a more nuanced analysis of the geometry of the data and capturing of the underlying dynamics of the time series. This study aims to verify the detection accuracy for changes in mean and variance within time-series data using simulated data and to confirm the precision in detecting apnea events using real-world data, as well as under conditions of strong noise interference. This will ascertain whether enhanced accuracy can be achieved in response to realistic noise scenarios that are likely to occur.

The remainder of this paper is organized as follows. The Methods section reviews change-point detection using the sub-sequence data and defines the proposed method. The Experimental Results and Discussion section evaluates the proposed method using two artificial datasets and real-world contact-free sensor data. Finally, the Conclusions section presents the conclusions and future work of the study.

## Methods

This section establishes the problem and formulates change-point detection using sub-sequence data. Furthermore, the SST method based on the proposed method is introduced. Finally, the proposed change-point detection method is described.

### Problem setup

**Fig 1:**
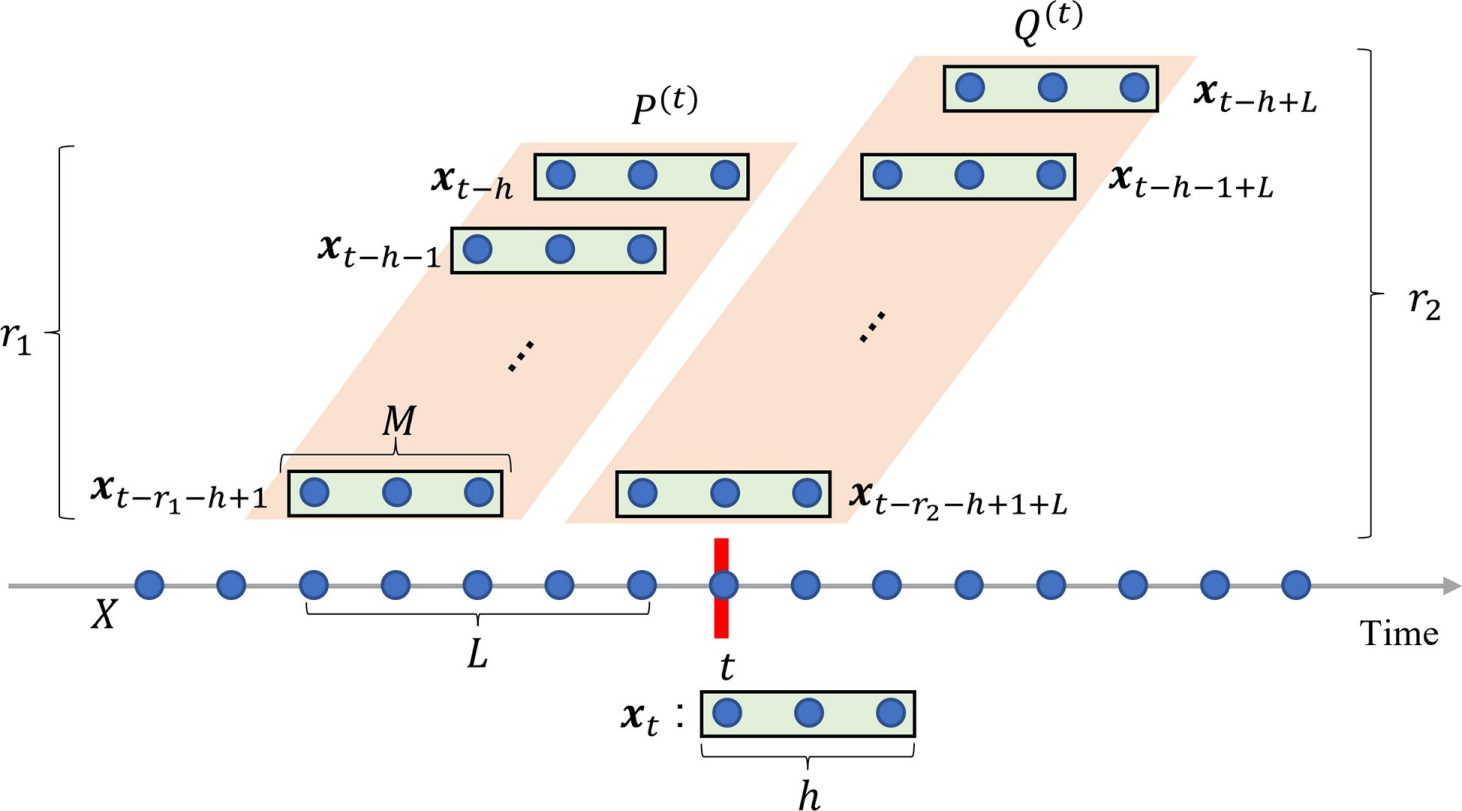
Example of notations in one-dimensional time-series data. *x*_*t*_ is the data point at time *t* with consecutive points *h*. Two windows, *P*^(*t*)^ and *Q*^(*t*)^, are shown at different positions along the time axis, each containing a subset of the data points within lengths *r*_1_ and *r*_2_, respectively. *L* represents the lag between the window *P*^(*t*)^ and *Q*^(*t*)^.

Suppose that *X* = [***x***_**1**_,***x***_**2**_,⋯,***x***_***N***_]∈ℝ^*N*^ is the one-dimensional (1D) sequence data of *N* time points, and ***x***_*t*_ = [*x*_*t*_,*x*_*t*+1_,⋯,*x*_*t+h*_]∈ℝ^*h*^ consists of *h* consecutive data points starting at time *t*. The parameter *h* is called the window size. Therefore, the past sub-sequence data *P*^(*t*)^ and current sub-sequence data *Q*^(*t*)^ can be defined as follows:

$$P^{\left(t\right)}=[x_{t-r_{1}-h+1},\ldots ,x_{t-h-1},x_{t-h}]=[p_{1},p_{2},\ldots ,p_{r_{1}}]\in R^{h\times r_{1}},$$ (1)

$$Q^{\left(t\right)}=[x_{t-r_{2}-h+1},\ldots ,x_{t-h-1+L},x_{t-h+L}]=[q_{1},q_{2},\ldots ,q_{r_{2}}]\in R^{h\times r_{2}},$$ (2)

where *r*_1_ and *r*_2_ represent the number of vectors *x*_*t*_ aggregated in matrices *P*^(*t*)^ and *Q*^(*t*)^, respectively; and *L* is a non-negative constant that indicates the time difference between *P*^(*t*)^ and *Q*^(*t*)^, which is referred to as the lag. Notably, *P*^(*t*)^ and *Q*^(*t*)^ are the Hankel matrices that serve a function in changepoint detection using subspace learning [29–31, 33]. Fig 1 illustrates an example of the notations used for 1D sequence data.

In change-point detection using sub-sequence time-series data, subspaces are obtained from *P*^(*t*)^ and *Q*^(*t*)^, and the score of the difference between subspaces is defined as the change score. If the change score at time *t* is greater than the threshold value, it is detected as a change point.

### Singular Spectrum Transformation (SST)

SST involves performing PCA to obtain the subspace from the generated sub-sequence data *P*^(*t*)^ and *Q*^(*t*)^. Essentially, it computes the $\hat{r}$ left singular vector $U_{\hat{r}}^{\left(t\right)}\in R^{w\times \hat{r}}$ and $Z_{\hat{r}}^{\left(t\right)}\in R^{w\times \hat{r}}$ by singular-value decomposition of matrices *P*^(*t*)^ and *Q*^(*t*)^, respectively.

$$P^{\left(t\right)}\approx U_{\hat{r}}^{\left(t\right)}\Sigma^{P}{V^{P}}^{T},$$

$$Q^{\left(t\right)}\approx Z_{\hat{r}}^{\left(t\right)}\Sigma^{Q}{V^{Q}}^{T},$$

where Σ is the diagonal matrix with singular values as diagonal elements and *V* is the right singular vector. The change in score is defined as follows:

$$score=1-{\left\|{U_{r}^{\left(t\right)}}^{T}Z_{r}^{\left(t\right)}\right\|}_{2}.$$

Singular-value decomposition performed in SST may result in insufficient change-point detection for waveforms with a nonlinear structure owing to the linear dimensionality-reduction method. This method was proposed for multichannel (multidimensional) sequence data [34].

### Proposed method (CPD-DM)

**Fig 2:**
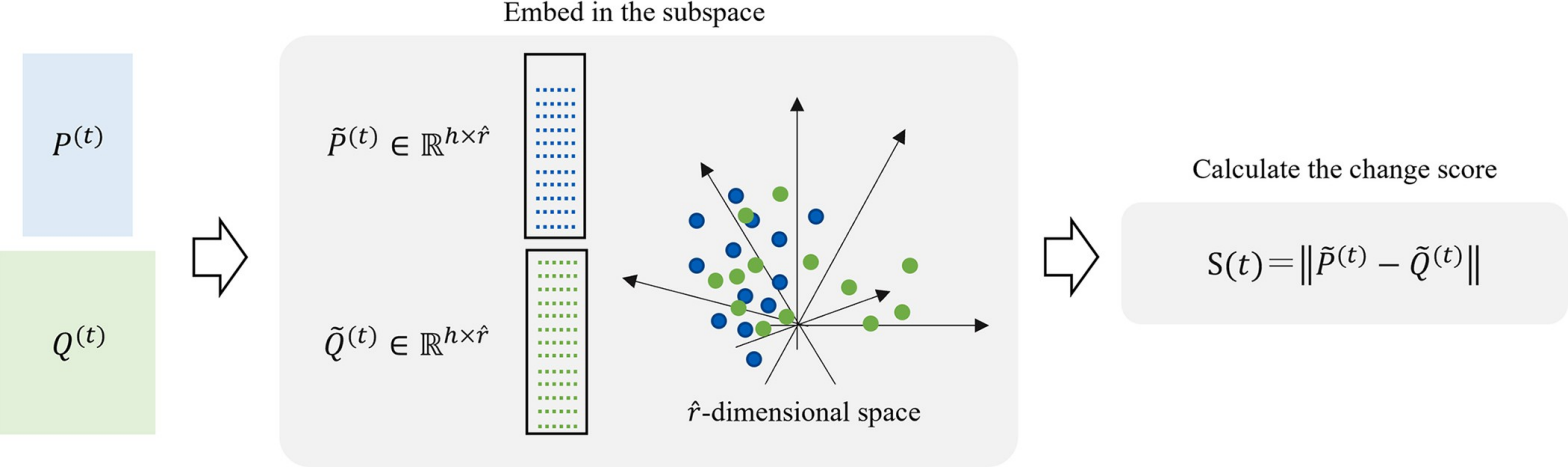
Framework of the proposed CPD-DM method. *P*^(*t*)^ and *Q*^(*t*)^ are transformed into their respective ${\tilde{P}}^{\left(t\right)}$ and ${\tilde{Q}}^{\left(t\right)}$ representation in an $\hat{r}$-dimensional space. The change score *S*(*t*) is calculated by measuring the norm of the difference between ${\tilde{P}}^{\left(t\right)}$ and ${\tilde{Q}}^{\left(t\right)}$.

The overall framework of the CPD-DM is depicted in Fig 2. This framework is based on SST and comprises two parts. First, the subspace is determined using a diffusion map from each of the two sub-sequence data *P*^(*t*)^ and *Q*^(*t*)^. Next, change points are extracted using the difference in the space as a change score. Furthermore, an extension of the proposed method for application in multidimensional sequence data is discussed below.

#### (a) Embedding in the subspace

The current sub-sequence data *Q*^(*t*)^ was embedded in the same subspace after determining the low-dimensional space using the diffusion map for past sub-sequence data *P*^(*t*)^. The diffusion map defines a distance function called the diffusion distance, which corresponds to the geodesic distance on a manifold, by defining a random walk on a set of *M* data points and constructing a low-dimensional space in which this distance is equal to the Euclidean distance.

First, a graph of data *P*^(*t*)^ is constructed. Next, the Gaussian kernel function is used for the edge weight of the graph. The *i*,*j* elements of the matrix *W* are as follows:

$$w_{i,j}=\exp\left(-\frac{{\left\|p_{i}-p_{j}\right\|}_{2}^{2}}{\sigma_{i}\;\sigma_{j}}\right),$$

where *σ*_*i*_ and *σ*_*j*_ are the local scale parameters for ***p***_*i*_ and ***p***_*j*_, respectively. Here, $\sigma_{i}={\left\|p_{i}-p_{j}^{K}\right\|}_{2}$, where $p_{j}^{K}$ is the *K*th nearest neighbor of ***p***_*i*_. Notably, Zelink–Manor and Perona [35] recommended a *K* value of 7, which was adopted in this study. Next, matrix *W* is normalized such that the rows sum to 1. Therefore, matrix *D*^(1)^ is formed, as follows.


$$d_{i,j}^{\left(1\right)}=\frac{w_{i,j}}{\sum_{k}w_{i,k}}.$$

Matrix *D*^(1)^ is considered a Markov matrix that defines the forward transition probability matrix of the dynamical process, because the diffusion map originates from the dynamical system theory. Therefore, matrix *D*^(1)^ represents the probability of transition from one data point to another in a single time step. The forward probability matrix of *α* time step *D*^(*α*)^ is given by ${\left(D^{\left(1\right)}\right)}^{\alpha }$. Using the random walk forward probability $d_{i,j}^{\left(\alpha \right)}$, the diffusion distance is defined as follows.

$${Diffusion}^{\left(\alpha \right)}(p_{i},p_{j})=\sqrt{\sum_{k}\frac{{\left(d_{i,k}^{\left(\alpha \right)}-d_{j,k}^{\left(\alpha \right)}\right)}^{2}}{\psi {\left(p_{k}\right)}^{\left(0\right)}}},\;\psi {\left(p_{i}\right)}^{\left(0\right)}=\frac{b_{i}}{\sum_{j}b_{j}},$$

where *b*_*i*_ is the degree of node ***p***_*i*_ defined by *b*_*i*_ = *Σ*_*j*_*d*_*i*,*j*_. With the spectral theory of random walk (refer to [36]), the representation ${\tilde{P}}^{\left(t\right)}$ that ensures that the low-dimensional diffusion distance *Diffusion*^(*α*)^(***p***_*i*_,***p***_*j*_) is as good as possible is formed by the $\hat{r}$ eigenvectors of the eigenproblem

$$D^{\left(\alpha \right)}\phi_{i}=\lambda_{i}\phi_{i}.$$ (3)

In a low-dimensional representation, the eigenvector is normalized by the corresponding eigenvalue, that is,

$${\tilde{P}}^{\left(t\right)}=[\lambda_{1}\phi_{1},\lambda_{2}\phi_{2},\ldots ,\lambda_{\hat{r}}\phi_{\hat{r}}],$$ (4)

where $\hat{r}$ is the reduced dimension.

Finally, the low-dimensional representation ${\tilde{Q}}^{\left(t\right)}=[{\tilde{q}}_{1},{\tilde{q}}_{2},\ldots ,{\tilde{q}}_{\hat{r}}]$ is determined by projecting the current sub-sequence data *Q*^(*t*)^ to the same subspace as ${\tilde{P}}^{\left(t\right)}$, and the linear mapping is calculated using a nonparametric out-of-sample extension method [37]; notably, this method can be applied to all nonlinear dimensionality-reduction methods. Therefore, the nearest neighbor of the data point ***q***_*i*_ in the original dimensional space is determined, and linear mapping from the nearest neighbor to the corresponding low-dimensional space is calculated. The low-dimensional space ${\tilde{Q}}^{\left(t\right)}$ is represented by applying linear mapping to the data points of *Q*^(*t*)^.

#### (b) Definition of change score

In the same embedded subspace, the score *S*(*t*) at time point *t* is defined as the distance between matrices ${\tilde{P}}^{\left(t\right)}$ and ${\tilde{Q}}^{\left(t\right)}$, as follows:

$$S(t)=\|{\tilde{P}}^{\left(t\right)}-{\tilde{Q}}^{\left(t\right)}\|.$$ (5)

The embedded low-dimensional space considers the global structure, while preserving the local data structure in the original dimensional space. Therefore, if the local and global data structures in the original dimensional space of the two sub-sequence data *P*^(*t*)^ and *Q*^(*t*)^ are similar, they are embedded close to each other in the low-dimensional space. Consequently, when the score is small, the structures of *P*^(*t*)^ and *Q*^(*t*)^ are similar; in contrast, when it is large, a large change in *P*^(*t*)^ and *Q*^(*t*)^ is observed.

#### (c) Extension to multidimensional data

For multidimensional data, the past and current sub-sequence data *P* and *Q* are set as follows:

$$P=[P_{1}^{\left(t\right)},P_{2}^{\left(t\right)},\ldots ,P_{m}^{\left(t\right)}]\in R^{h\times (r_{1}m)},$$ (6)

$$Q=[Q_{1}^{\left(t\right)},Q_{2}^{\left(t\right)},\ldots ,Q_{m}^{\left(t\right)}]\in R^{h\times (r_{2}m)},$$ (7)

where $P_{i}^{\left(t\right)}$ and $Q_{i}^{\left(t\right)}$ are calculated using Eq (1) and (2), respectively, for *i*th dimension data. Next, steps (a) and (b) are executed for matrices *P* and *Q*. Algorithm 1 summarizes the algorithm for the proposed collaborative novelty detection.

 **Algorithm 1.**

**Input: S**equence data *X*∈ℝ^*N*×*m*^, dimension of the reduced space $\hat{r}$, window size *h*, and lag *L*.

**Output: change score *S*.**

**For each time point:**

 1. Calculate the sub-sequence matrices *P* and *Q* in Eqs (6) and (7) from the current and past sub-sequence data.

 2. Solve Eq (3) for eigenvalues and eigenvectors from matrix *P*.

 3. Compute the low-dimensional representation ${\tilde{P}}^{\left(t\right)}$ in Eq (4).

 4. Compute the low-dimensional space ${\tilde{Q}}^{\left(t\right)}$ by applying linear mapping.

 5. Compute the change score *S*(*t*).

## Experimental results and discussion

This section evaluates the proposed CPD-DM for synthetic and contact-free sensor datasets. The proposed method was compared with conventional statistical methods, namely, the H-T2, *k*-nearest neighbor (KNN), and SST methods. The area under the curve (AUC), F1-score, and Rand index (RI) [38] were used as the performance metrics for the detected change points. Let *n* be the number of correct change points at moments *τ*_1_,*τ*_2_,⋯,*τ*_*n*_ and $\hat{n}$ be the number of predicted change points at moments ${\hat{\tau }}_{1},{\hat{\tau }}_{2},\ldots ,{\hat{\tau }}_{\hat{n}}$. A set of correctly detected change points is defined as the true positive (TP) [39]: $TP=\{\tau_{i}|\exists {\hat{\tau }}_{j}:|{\hat{\tau }}_{j}-\tau_{i}|<M\}$, where *M* is a margin size. The F1-score measure is then defined as follows:

$$F1=\frac{2∙Precision∙Recall}{Precision+Recall},$$

where $Precision=|TP|/\hat{n}$, and *Recall* = |*TP*|/*n*. For the AUC, a binary classification problem is considered, where the true label consists of {0,1}, class 1 contains the correct change-points in $\left[\tau_{i}-M\leq \tau_{i}\leq \tau_{i}+M](i=1,2,\ldots ,n\right)$, and class 0 the rest. Subsequently, the AUC is calculated using the true labels and change-point scores. Notably, F1 and RI determine the number of change points to be detected. Next, the detected points are checked to ascertain whether they fall within *M* points before and after the true change point. The AUC uses the magnitude of the change score to calculate the detection measurements without determining the number. Here, the optimal value of the embedded dimensions was selected as 1, 2, …, 10 for CPD-DM and SST, time step *α* as 0.1, 0.5, 1, and 10 for CPD-DM, nearest neighbors as 1,2,⋯,10 for KNN, and window size as 20, 40, …, 200 for all methods to achieve the best AUC.

### Synthetic datasets

In this study, two types of toy problems were considered. For both dataset types, 10 samples of 10D data were created, with a length of 2000, and a change-point was generated every 200-time points. The first dataset, denoted as (a), contained data in which the average value changed, and the second dataset, denoted as (b), contained data in which the variance value changed. Next, [0, 1] range normalization was performed for preprocessing, the window size *h* was set to 20, 40, …, and 200 for all methods to achieve the best AUC, lag $L=\frac{h}{4}$, and margin *M* = 20.

**Fig 3:**
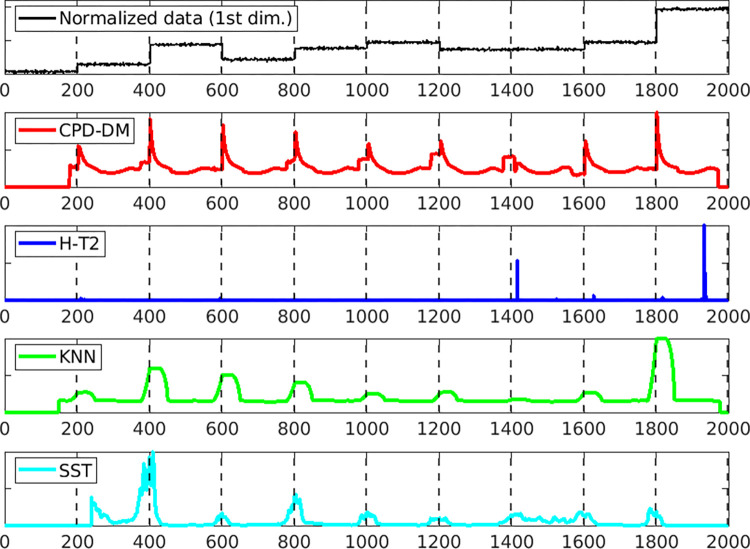
Change scores of each method for one sample of (a) average value changes dataset. The top panel shows the normalized data along the first dimension of average value changes dataset. Subsequent panels illustrate the change scores obtained using the proposed method (CPD-DM; red), Hotelling’s *T*^2^ (H-T2; blue), *k*-nearest neighbor (KNN; green), and singular spectrum transformation (SST; cyan). Each method’s change scores are plotted over the same time scale, with significant peaks indicating detected change points. The vertical dashed lines represent the actual change points.

**Fig 4:**
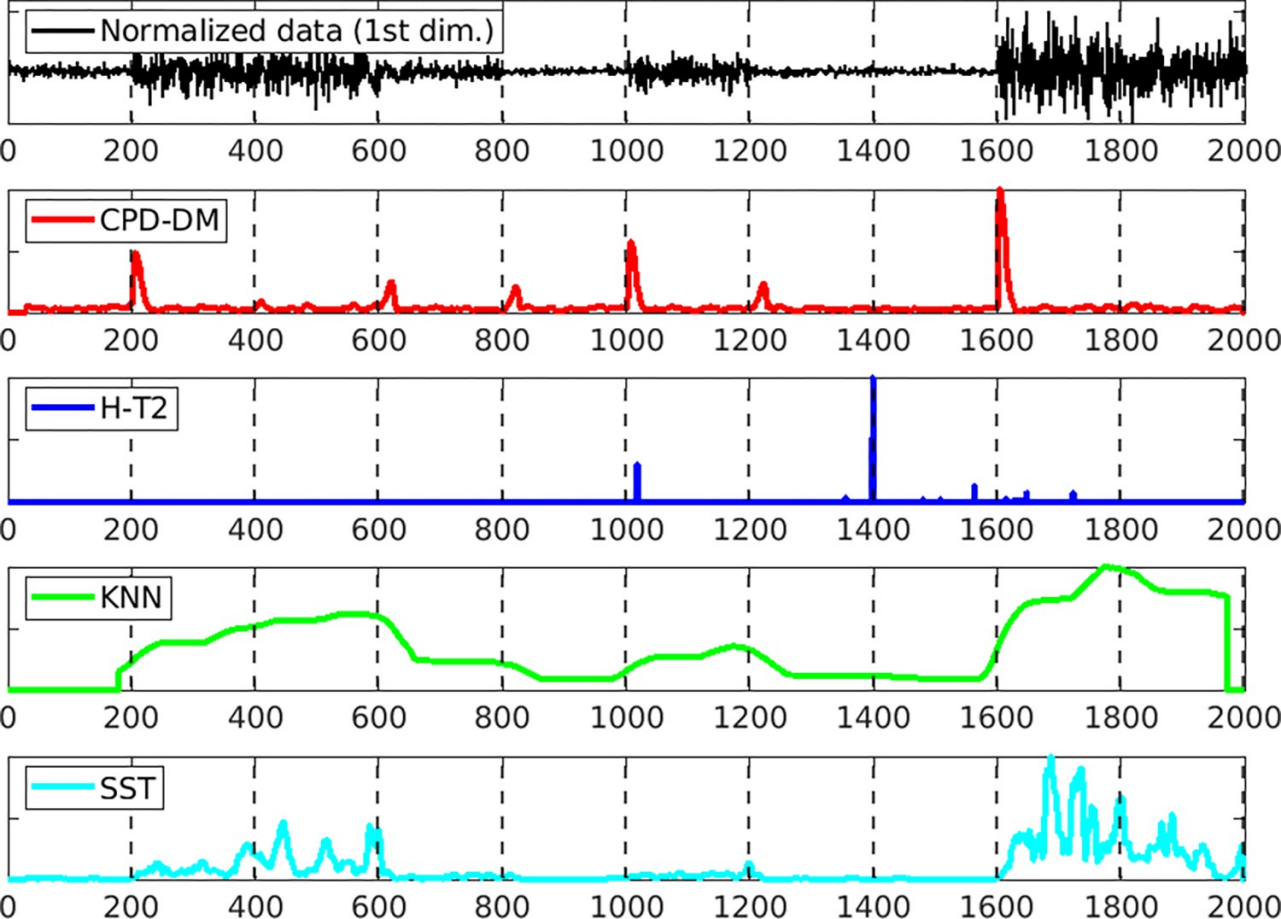
**Change scores of each method for one sample of (b) variance value changes dataset.** The top panel shows the normalized data along the first dimension of variance value changes dataset. Subsequent panels illustrate the change scores obtained using the proposed method (CPD-DM; red), Hotelling’s *T*^2^ (H-T2; blue), *k*-nearest neighbor (KNN; green), and singular spectrum transformation (SST; cyan). Each method’s change scores are plotted over the same time scale, with significant peaks indicating detected change points. The vertical dashed lines represent the actual change points.

Figs 3 and 4 illustrate the change score for each method depending on the window size when the AUC is the best for both datasets. The top image shows the first dimension of the normalized data, and the dotted lines indicate the changepoints. Evidently, the proposed CPD-DM detected almost all the change points, and the grater the change, the larger the change score, such as 400, 600, and 1800 points for dataset (a) and 200, 1000, and 1600 points for dataset (b). In dataset (a), the KNN and SST exhibited low change scores at points with small changes, which were difficult to detect as change points. In dataset (b), the KNN, SST, and H-T2 methods barely detected the true change points.

Table 1 presents the mean values of AUC, F1, and RI for datasets (a) and (b). The bold numbers are the best scores. In dataset (a), the proposed method was the best for all measures. In dataset (b), the proposed method obtained the best results in terms of the AUC. For the F1 measure, H-T2 yielded the best values; however, all the methods exhibited low accuracy. For the RI measure, the KNN and SST achieved the best values; however, the accuracy of the proposed CPD-DM was similar. Therefore, CPD-DM’s change scores are highly reliable when detecting the change points, making it more accurate with fewer false positives than the other methods.

**Table 1:** Mean and standard deviations (SDs) of performance metrics for two artificial datasets.

**(a) Average value changes**			
	**AUC**	**F1**	**RI**
**CPD-DM**	**0.851 ± 0.037**	**0.642 ± 0.078**	**0.925 ± 0.004**
**H-T2**	0.785 ± 0.010	0.347 ± 0.141	0.775 ± 0.127
**KNN**	0.839 ± 0.010	0.105 ± 0.000	0.920 ± 0.000
**SST**	0.808 ± 0.011	0.232 ± 0.044	0.769 ± 0.114
**(b) Variance value changes**			
	**AUC**	**F1**	**RI**
**CPD-DM**	**0.665 ± 0.030**	0.179 ± 0.071	0.915 ± 0.005
**H-T2**	0.593 ± 0.010	**0.242 ± 0.112**	0.735 ± 0.140
**KNN**	0.542 ± 0.002	0.105 ± 0.000	**0.920 ± 0.000**
**SST**	0.536 ± 0.025	0.116 ± 0.060	**0.920 ± 0.005**

The metrics provided as the area under the curve (AUC), F1-score, and Rand index (RI) are used to evaluate the effectiveness of the proposed method (CPD-DM), Hotelling’s *T*^2^ (H-T2), *k*-nearest neighbor (KNN), and singular spectrum transformation (SST) methods in detecting change points within (a) average value changes dataset and (b) variance value changes dataset.

### Contact-free sensor signals

A public dataset [17] obtained from previous research was used in this study. It was collected from 30 healthy volunteers who performed an apnea hemodynamic scenario. However, this dataset was not recorded for all the subjects, and consequently, it contains data of only 23 subjects. In this scenario, the participants held their breath twice for as long as possible. In the first simulation, they fully inhaled before holding their breath. In the second simulation, they fully exhaled before holding their breath. Because the button was pressed at the same time as the event in the simulation, the times when breath-holding began and ended were given. In this study, we used the start and end points of the simulation as change points. Three features from the contact-free sensors were employed: radar I, radar Q, and distance (computed from radar I and radar Q). For data preprocessing, the absolute value of the fast Fourier transformation for each feature was added; that is, six features were used. The original data were sampled at 2000 frequencies and initially downsampled to a sampling frequency of 100 Hz. The data length ranged from 9550–40180. Furthermore, the window size *h* was set to 200, 400, …, 2000 for all the methods to achieve the best AUC, and $L=\frac{h}{4}$ and *M* = 500 were utilized as performance metrics. An experiment was performed by adding noise to the contact-free sensors to confirm the robustness of the proposed method against noise: the noise was a random number generated from a normal distribution with a mean of 0 and an SD of $\hat{\sigma }$. Moreover, an experiment was performed by varying $\hat{\sigma }$ from 0.1 to 1, in increments of 0.1.

Table 2 presents the mean performance metrics without the noise dataset. The bold values are the best scores. The proposed CPD-DM exhibited the best performance in detecting the change points for contact-free sensor data. For the AUC, H-T2 yielded the worst performance, and KNN and SST exhibited acceptable performance but were inferior to CPD-DM. Therefore, the proposed method can detect the point at which respiration stops more accurately than the conventional methods in an apnea scenario using contact-free sensor data.

**Table 2:** Mean and SDs of performance measures without noise data.

	AUC	F1	RI
**CPD-DM**	**0.728 ± 0.075**	**0.434 ± 0.219**	**0.799 ± 0.070**
**H-T2**	0.497 ± 0.089	0.213 ± 0.156	0.743 ± 0.079
**KNN**	0.725 ± 0.097	0.172 ± 0.195	0.733 ± 0.081
**SST**	0.708 ± 0.079	0.346 ± 0.159	0.785 ± 0.064

The table includes the area under the curve (AUC), F1-score, and Rand index (RI) for the proposed method (CPD-DM), Hotelling’s *T*^2^ (H-T2), *k*-nearest neighbor (KNN), and singular spectrum transformation (SST) methods.

**Fig 5:**
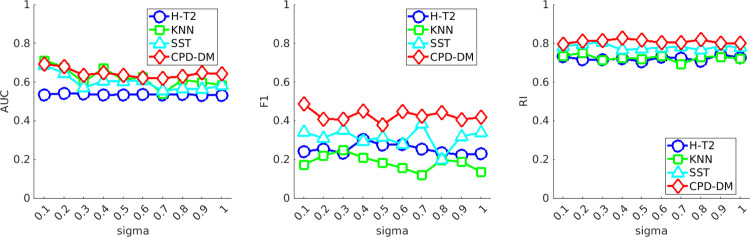
Mean performance measures for changing standard deviations of noise. Area under the curve (AUC), F1-score, and Rand index (RI) for each change point detection method under varying levels of noise standard deviation (sigma). The performance of the proposed method (CPD-DM; red), Hotelling’s *T*^2^ (H-T2; blue), *k*-nearest neighbor (KNN; green), and singular spectrum transformation (SST; cyan) is plotted to illustrate the effect of noise on detection accuracy. Each line connects the mean metric values calculated at different sigma values, demonstrating the robustness of the methods to noise.

Fig 5 depicts the performance metrics for the sensor dataset with noise when the SD is varied from 0.1 to 1 in increments of 0.1. The proposed CPD-DM achieved performance measure values equivalent to or higher than those of the conventional methods. In terms of the AUC, KNN and SST could not maintain their accuracy when the SD of the noise increased, that is, when the noise increased. However, the proposed method maintained its accuracy under this condition. Therefore, the proposed CPD-DM is robust against noise.

Because this experiment can be verified with only one dataset, more diverse data sources were necessary. In the future, the performance of the proposed method in real-world scenarios, such as monitoring of patients with sleep apnea using contact-free sensors in home or hospital settings, should be investigated. Moreover, the scalability and computational efficiency of this method need to be evaluated when dealing with large data and various sensor types. The experimental results provided evidence to support the hypothesis regarding the effectiveness of the proposed change-point detection method using diffusion maps for apnea simulations. The results demonstrate that the method outperforms the conventional SST method in terms of accuracy and robustness against noise. Furthermore, the findings suggest that the proposed method cab be applied to other contactless sensor data types and that its potential usefulness can be extended beyond sleep monitoring. The development of novel methods that can deal with complex nonlinear structures can improve the accuracy of change-point detection in various applications, including sleep monitoring and other health-related monitoring tasks.

## Conclusions

In this study, we proposed a novel change-point detection method to accurately detect the change points (i.e., respiration stopping points). Our method utilizes both, current and past sub-sequence data, at a point and embeds them in a low-dimensional space using diffusion maps. Because the global structure is considered in the low-dimensional space while preserving the local data structure in the original space, the distance between two sub-sequence data is used as the change score. A large distance indicates that the preceding and following sub-sequence data at a point are not similar and the change is significant. Furthermore, the generalization of multidimensional signal data was proposed. Numerical tests verified the effectiveness of the proposed method. Experiments were conducted using synthetic and real-world contact-free sensor data to evaluate the performance of the methods under various scenarios, including the presence of noise or other common obstacles found in the sensor data. We confirmed that the proposed method was robust to noise, accurately detected change points in the simulation of sleep apnea, and was superior to conventional methods in terms of accuracy and reliability. Furthermore, the proposed method reduces the testing load, supports monitoring in various settings, and ensures privacy. In the future, the generalizability of this method to other types of data should be explored and its performance in real-world applications should be evaluated. Furthermore, the scalability of the method and its computational efficiency in handling large-scale data must be considered. This study contributes to improving sleep monitoring by providing a more accurate and reliable change-point detection method. The proposed method can also be applied to other fields such as healthcare, manufacturing, and finance where change-point detection is important. Ultimately, this research will contribute to the development of advanced monitoring systems that guarantee privacy and adaptability across various scenarios.

## References

[1] Sigurdson K, and Ayas NT. The public health and safety consequences of sleep disorders. Can J Pharmacol. 2007;85(1): 179–183. doi: 10.1139/y06-095 17487258

[2] Medic G, Wille M, Hemels ME. Short- and longterm health consequences of sleep disruption, Nat Sci Sleep. 2017; 151–161. doi: 10.2147/NSS.S134864 28579842 PMC5449130

[3] Könen T, Dirk J, Schmiedek F. Cognitive benefits of last night’s sleep: daily variations in children’s sleep behavior are related to working memory fluctuations. J Child Psychol Psychiatry. 2015;56(2):171–82. doi: 10.1111/jcpp.12296 25052368

[4] Pilcher JJ, Ginter DR, Sadowsky B. Sleep quality versus sleep quantity: relationships between sleep and measures of health, well-being and sleepiness in college students. J Psychosom Res. 1997;42(6):583–96. doi: 10.1016/s0022-3999(97)00004-4 9226606

[5] Lentino CV, Purvis DL, Murphy KJ, Deuster PA. Sleep as a component of the performance triad: the importance of sleep in a military population. US Army Medical Department Journal. 2013. 24146247

[6] Bower B, Bylsma LM, Morris BH, Rottenberg J. Poor reported sleep quality predicts low positive affect in daily life among healthy and mood-disordered persons. J Sleep Res. 2010;19:323–332. doi: 10.1111/j.1365-2869.2009.00816.x 20374447

[7] Van der Helm E, Walker MP. The role of sleep in emotional brain regulation. In: Kring AM, Sloan DM, editors. Emotion regulation and psychopathology: A transdiagnostic approach to etiology and treatment. New York, NY US: Guilford Press; 2010. pp. 253–279.

[8] John A, Nundy KK, Cardiff B, John D. SomnNET: An SpO2 Based Deep Learning Network for Sleep Apnea Detection in Smartwatches. 2021 43rd Annual International Conference of the IEEE Engineering in Medicine and Biology Society (EMBC), 2021. pp. 1961–1964. 10.1109/EMBC46164.2021.9631037 34891671

[9] Peng X, Dong K, Ning C, Cheng R, Yi J, Zhang Y, et al. All-nanofiber self-powered skin-interfaced real-time respiratory monitorng system for obstructive sleep apnea-hypopnea syndrome diagnosing. Adv Funct Mater. 2021:31(34): 2103559. doi: 10.1002/adfm.202103559

[10] Medical Advisory Secretariat. Polysomnography in patients with obstructive sleep apnea: an evidence-based analysis. Ont Health Technol Assess Ser. 2006;6(13):1–38. Epub 2006 Jun 1. 23074483 PMC3379160

[11] Caples SM, Anderson WM, Calero K, Howell M, Hashmi SD. Use of polysomnography and home sleep apnea tests for the longitudinal management of obstructive sleep apnea in adults: an American Academy of Sleep Medicine clinical guidance statement. J Clin Sleep Med. 2021;17(6):1287–1293. doi: 10.5664/jcsm.9240 33704050 PMC8314660

[12] Madsen S, Baczuk J, Thorup K, Barton R, Patwari N, Langell JT. A noncontact RF-based respiratory sensor: results of a clinical trial. J Surg Res. 2016;203(1):1–5, doi: 10.1016/j.jss.2016.03.018 27338527

[13] Schellenberger S, Shi K, Steigleder T, Malessa A, Michler F, Hameyer L, et al. A dataset of clinically recorded radar vital signs with synchronised reference sensor signals. Sci Data. 2020;7: 291. doi: 10.1038/s41597-020-00629-5 32901032 PMC7479598

[14] Cardillo E, Caddemi A. Radar range-breathing separation for the automatic detection of humans in cluttered environments. IEEE Sens J. 2021;21: 14043–14050. doi: 10.1109/JSEN.2020.3024961

[15] Su WC, Tang MC, Arif RE, Horng TS, Wang FK. Stepped-frequency continuous-wave radar with self-injection-locking technology for monitoring multiple human vital signs. IEEE Trans Microw Theory Tech. 2019;67: 5396–5405. doi: 10.1109/TMTT.2019.2933199

[16] Su WC, Huan PH, Chian DM, Horng TSJ, Wen CK. Wang FK. 2-D self-injection-locked doppler radar for locating multiple people and monitoring their vital signs. IEEE Trans Microw Theory Tech. 2021;69: 1016–1026. doi: 10.1109/TMTT.2020.3037519

[17] Wang X, Yang C, Mao S. Tensor decomposition for monitoring multiperson breathing beats with commodity wifi. ACM Trans Intell Syst Technol (TIST). 2017;9: 1–27. doi: 10.1145/3078855

[18] Abdelnasser H, Harras KA, UbiBreathe YM. A ubiquitous non-invasive wifi-based breathing estimator. In: Proceedings of the 16th ACM International Symposium on Mobile Ad Hoc Networking and Computing; 2015. pp. 277–286.

[19] Liu J, Wang Y, Chen Y, Yang J, Chen X, Cheng J. Tracking vital signs during sleep leveraging off-the-shelf wifi. In: Proceedings of the 16th ACM International Symposium on Mobile Ad Hoc Networking and Computing; 2015. pp. 267–276. doi: 10.1145/2746285.2746303

[20] Bhattacharya A, Vaughan R. Deep learning radar design for breathing and fall detection. IEEE Sens J. 2020;20: 5072–5085. doi: 10.1109/JSEN.2020.2967100

[21] Zhao H, Hong H, Miao D, Li Y, Zhang H, Zhang Y, et al. A Noncontact breathing disorder recognition system using 2.4-ghz digital-if doppler radar. IEEE J Biomed Health Inform. 2019;23: 208–217. doi: 10.1109/JBHI.2018.2817258 29993789

[22] Basu S, Meckesheimer M. Automatic outlier detection for time series: An application to sensor data. Knowl Inf Syst. 2006;11: 137–154. doi: 10.1007/s10115-006-0026-6

[23] Reeves J, Chen J, Wang XL, Lund R, Lu QQ. A review and comparison of changepoint detection techniques for climate data. J Appl Meteorol Climatol. 2007;46: 900–915. doi: 10.1175/JAM2493.1

[24] Rybach D, Gollan C, Schluter R, Ney H. Audio segmentation for speech recognition using segment features. Proc IEEE Int Conf Acoust Speech Signal Process. 2009. pp. 4197–4200. doi: 10.1109/ICASSP.2009.4960554

[25] Page ES. Continuous inspection schemes. Biometrika. 1954;41: 100–115. doi: 10.2307/2333009

[26] Lee S, Ha J, Na O, Na S. The cusum test for parameter change in time series models. Scand Stat Theory Appl. 2003;30: 781–796. doi: 10.1111/1467-9469.00364

[27] Shao X, Zhang X. Testing for change points in time series. J Am Stat Assoc. 2010;105: 1228–1240. doi: 10.1198/jasa.2010.tm10103

[28] Yau CY, Zhao Z. Inference for multiple change points in time series via likelihood ratio scan statistics. J R Stat Soc B. 2016;78: 895–916. doi: 10.1111/rssb.12139

[29] Moskvina V, Zhigljavsky AA. Application of the singular-spectrum analysis to changepoint detection in time series. Seq Anal. 2003a.

[30] Ide T, Tsuda K. Change-point detection using Krylov subspace learning. In: Proceedings of the Siam International Conference on Data Mining; 2007. pp. 515–520.

[31] Itoh N, Kurths J J. Change-point detection of climate time series by nonparametric method. In: Proceedings of the World Congress on Engineering and Computer Science; 2010. pp. 445–448.

[32] Coifman RR, Lafon S. Diffusion maps. Appl Comput Harman Anal. 2006:21(1): 5–30. doi: 10.1016/j.acha.2006.04.006

[33] Kawahara Y, Yairi T, Machida K. Change-point detection in time-series data based on subspace identification. In: Proceedings of the 7th IEEE International Conference on Data Mining (ICDM 2007); 2007. pp. 559–564. doi: 10.1109/ICDM.2007.78

[34] Tokunaga T, Ikeda D, Nakamura K, Higuchi T, Yoshikawa A, Uozumi T, et al. Detecting precursory events in time series data by an extension of singular spectrum transformation. In: Proceedings of the 10th WSEAS International Conference on Applied Computer Science; 2010. pp. 366–374.

[35] Manor LZ, Perona P. Self-tuning spectral clustering. Proceedings of the Advances in Neural Information Processing Systems. 2005;27: 1601–1608.

[36] Lafon S, Lee AB. Diffusion maps and coarse-graining: A unified framework for dimensionality reduction, graph partitioning, and data set parameterization. IEEE Trans Pattern Anal Mach Intell. 2006;28: 1393–1403. doi: 10.1109/TPAMI.2006.184 16929727

[37] Li H, Teng L, Chen W, Shen IF. Supervised Learning on local tangent space. Lect NotesComput Sci. 2005;3496: 546–551. doi: 10.1007/11427391_87

[38] Prates LO. A more efficient algorithm to compute the rand Index for change-point problems. Arxiv:2112.03738 [Preprint] 2021. [Cited 2023 Nov 1]. Available from: https://arxiv.org/abs/2112.03738.

[39] Hushchyn M, Arzymatov K, Derkach D. Online neural networks for change-point detection. Arxiv:2010.01388 [Preprint] 2020. [Cited 2023 Nov 1]. Available from: https://arxiv.org/abs/2010.01388.

